# An Efficient and Lightweight Deep Learning Model for Human Activity Recognition Using Smartphones

**DOI:** 10.3390/s21113845

**Published:** 2021-06-02

**Authors:** Ankita, Shalli Rani, Himanshi Babbar, Sonya Coleman, Aman Singh, Hani Moaiteq Aljahdali

**Affiliations:** 1 Chitkara University Institute of Engineering and Technology, Chitkara University, Punjab 140401, India; ankitaanand2719@gmail.com (A.); himanshi.babbar@chitkara.edu.in (H.B.); 2 Faculty of Computing, Ulster University Northern Ireland, Coleraine BT52 1SA, Northern Ireland, UK; 3 Department of Computer Science and Engineering, Lovely Professional University, Punjab 144411, India; amansingh.x@gmail.com; 4 Faculty of Computing and Information Technology, King Abdulaziz University, Jeddah 37848, Saudi Arabia; Hmaljahdali@kau.edu.sa

**Keywords:** human activity recognition, deep learning, convolutional neural network, long-short term memory

## Abstract

Traditional pattern recognition approaches have gained a lot of popularity. However, these are largely dependent upon manual feature extraction, which makes the generalized model obscure. The sequences of accelerometer data recorded can be classified by specialized smartphones into well known movements that can be done with human activity recognition. With the high success and wide adaptation of deep learning approaches for the recognition of human activities, these techniques are widely used in wearable devices and smartphones to recognize the human activities. In this paper, convolutional layers are combined with long short-term memory (LSTM), along with the deep learning neural network for human activities recognition (HAR). The proposed model extracts the features in an automated way and categorizes them with some model attributes. In general, LSTM is alternative form of recurrent neural network (RNN) which is famous for temporal sequences’ processing. In the proposed architecture, a dataset of UCI-HAR for Samsung Galaxy S2 is used for various human activities. The CNN classifier, which should be taken single, and LSTM models should be taken in series and take the feed data. For each input, the CNN model is applied, and each input image’s output is transferred to the LSTM classifier as a time step. The number of filter maps for mapping of the various portions of image is the most important hyperparameter used. Transformation on the basis of observations takes place by using Gaussian standardization. CNN-LSTM, a proposed model, is an efficient and lightweight model that has shown high robustness and better activity detection capability than traditional algorithms by providing the accuracy of 97.89%.

## 1. Introduction to Human Activity Recognition

Today, the human assistance recognition system has been an essential part in the lives of human. It recognizes the human’s presence or the current state/activity/action that relies upon that information that is being received from the various sensors. This system is used to detect the human’s motion daily activities. A HAR system has major areas of applications, such as interaction among humans and computers, remote monitoring, military, healthcare, gaming, sports, security, and surveillance. This assistance system has other areas of wide applications, such as monitoring human’s special activities, monitoring sleep disorders, rehabilitation activities behavior recognition, fall detection, etc. [1]. For physical and mental growth, physical activities play a major role which leads to many body-related problems, such as diabetes, heart attack, etc. There exist two physical activity recognition categories: (a) vision-based and (b) non-vision based. The vision-based approach is limited to environments’ sensitivity of light and detects at low range. The non-vision approach is mainly used for human activity recognition that uses a different variety of sensors on wearable devices [2]. The collection of huge data for HAR is a challenging task. Finding appropriate data and then preparing it for the further process makes use of costly sensors and issues related to privacy and security becomes difficult to use the public dataset. The big challenge for HAR is the classification of time sequence task. A person’s movement can be predicted with the help of data acquired by the sensors and traditionally involves expertise in deep domain and methods to correctly design the features from the raw data so that a machine learning model can be fit into, from the signal processing method. At present, deep learning methods, such as Convolutional Neural Networks (CNN) and Recurrent Neural networks (RNN), are very much capable of and have even achieved higher state-of-the-art results and have enabled the automatic learning of the features from the raw sensor data. HAR is broadly classified into Sensor-based, Vision-based and multimodal categories [3].

1. **Sensor-based**—The first HAR approach contains a large number of sensor type technologies that can be worn on-body known as wearable sensors, ambient sensors, and, together, both will make hybrid sensors that help in measuring quantities of human body motion. Various opportunities can be provided by these sensor technologies which can improve the robustness of the data through which human activities can be detected and also provide the services based on sensed information from real-time environments, such as cyber-physical-social systems [4]; there is also a type of magnetic senors when embedded in smartphone can track the positioning without any extra cost [5].
2. **Vision-based**—RGB video and depth cameras being used to obtain human actions.
3. **Multimodal**—Sensors data and visual data are being used to detect human activities.

The evolvement of IoT is increasing rapidly. The major reason behind the evolvement is the compatibility of IoT with that of wearable sensors, network objects, and conventional networks [6]. For example, the body sensor nodes are one of the most essential technologies of IoT by integrating both body sensor systems and networks of the wireless detector. There are various techniques used for information extraction. Deep Learning (DL) is an artificial intelligence (AI) function that uses structure-based algorithms functions, like a brain, to conclude and extract the important data from the given datasets [7]. Deep Learning is an emerging field of research. The traditional machine learning approach requires feature extraction manually, but the deep learning approach performs automatic feature extraction. With the help of sensing devices, measured data were collected, results were analyzed, and a HAR system was developed.

### Contributions of the Paper

1. A deep learning neural network based on CNN and LSTM model is used to train and recognize the different phases of human activities which includes the pre-processing of data, collection of data, and extraction of features from UCI-HAR dataset.
2. Different deep learning algorithms are implemented to check the accuracy of the proposed model.
3. Each input image the convolutional method is applied, and the output of each input image is transferred to the LSTM as a single time step. Filter maps taken as a a most important hyperparameter can be used for mapping of the different portions of an image.
4. Transformation on the basis of observations is done by using Gaussian standardization. CNN-LSTM, the proposed model, is efficient and lightweight model and has shown high robustness and better activity detection capability than traditional algorithms by providing the accuracy of 97.89%.
5. Next, use of Deep Learning classifiers for attaining the better accuracy and comparison table of the deep learning models is presented for better understanding.

The rest of the paper is organized as: in Section 2, architecture of HAR is discussed, followed by deep learning in HAR in Section 3. Public datasets for HAR are discussed in Section 4, while CNN is discussed in Section 5. Proposed light model is presented in Section 6. Result analysis and discussions are given in Section 7, and concluded observations are given in Section 8.

## 2. Related Work

A Human Recognition System has various approaches, such as vision-based and sensor-based, which further categorized into wearables, object-tagged, dense sensing, etc. [1]. Before moving further, there also exist some design issues in HAR systems, such as selection of different types of sensors, data collection related set of rules, recognition performance, how much energy is consumed, processing capacity, and flexibility [8]. Keeping all these parameters in mind, it is important to design an efficient and lightweight human activity recognition model. A network for mobile human activity recognition has been proposed using long-short term memory approach for human activity recognition using triaxial accelerometers data [9]. Before moving further, discussion of wearable sensors is a must. Wearable Sensors include human physical activities, such as walking, sitting, standing, and climbing, on wearable sensors that can be recognized by HAR [10] as these are known as the body-worn sensors that can catch all the human activities and can be located on human limbs or clothes. There exist wearable devices, like watches and wristbands, but, sometimes, it is not feasible for critically ill patients. The physiological and functional data to be used in the field of sports and the healthcare domain can be captured by wearable devices. The wearable device’s hardware part has an inertial sensor containing a microcontroller, a three-axis accelerometer, and a gyroscope. Signals acquisition, signal normalization, and a feature learning method are being used in the software part of the activity recognition algorithm. In HAR, there also exist many wearable sensors, like Biosensors, Inertial Measurement Units, Smartphone Sensors, and Global Positioning Systems [4]. Both inertial sensors and embedded systems, along with the wearable devices, are being used for the recognition of various daily and sports activities.

Combination of inertial sensors with those of the embedded ones in wearable’s activity recognition has a major advantage as they can work without any external interference of such types of sensors, like camera, radar, or any infrared sensors, which are an initial requirement for the wearables [11]. A feature selection approach for the HAR system using smartphone is proposed having built-in accelerometer for measuring of motion or physical activities of a human and a gyroscope for adding an additional dimension to the information given by the accelerometer by tracking up the rotation [11]. The HAR system with sensors using Deep Learning Convolutional Neural Network when compared with other models gave the more accurate results [12]. A Convolutional Neural Network for the HAR system having triaxial accelerometers in smartphones achieved the higher accuracy results [13] but in sequential real-time situations CNN alone is not enough as it only works in case of pictures but the combination of LSTM and CNN gives more accurate results in recognition of human activities, such as walking, running, sitting, standing, laying, etc. The Ref. [14] achieved accuracy of about 97.64% using principle component analysis (PCA)-bidirectional long short term memory(LSTM) approach so that LSTM recursive neural networks can be trained for prediction of the identified activities performed in the datasets and by using PCA results in the reduction in the datasets number of dimensions of 12 activities.

Further, in Reference [15], a CNN-LSTM method is proposed for the HAR system on different types of datasets and achieved different accuracies. In addition, by using smartphone sensor and deep learning models, an efficient and robust human activity recognition system have been developed in which different deep learning models, such as artificial neural network, support vector machines, and deep belief networks, have been compared with there proposed model only, but more efficient and complex model also needed to support the real time environments. There are many supervised learning methods that are being used for activity recognition. A comparative study on the existing work is shown in Table 1.

## 3. Architecture of HAR

The problem of predicting of what a person is doing based on a trace of their movement using sensors can be solved by Human Activity Recognition. The Human Activity Recognition dataset available for the public use is the ‘Activity Recognition Using Smart Phones Dataset’ that made available in 2012.

The various steps that take place for the architecture of the following system are Collection of data and pre-processing the data, the next step is the feature extraction then Activity classification, and last is the Evaluation [8]. Data is collected from different sensors and pre-processing of that data takes place for future use. Data can be cleaned through various applications, such as raw signal representation, noise removal, and missing values can be dealt with. The data after being cleaned is differentiated into windows. Window fragmentation has different approaches, such as event-based window, sliding window, and energy [19]. The next step, Feature Extraction, is done to increase the efficiency of an algorithm to reduce the set of features. To do this, various approaches are there, such as traditional machine learning, deep learning, etc. Traditional Machine Learning extracts the features manually, but deep learning is used for automatic feature extraction. A classification algorithm is used for the features extracted, and there exist several metrics for performance evaluation, such as accuracy, confusion matrix, precision, f1-score, and recall [20].

### Modules of HAR

The following system has two modules, one of which is a traditional HAR system used on any mobile and non-mobile device, and other is an e-health application in the healthcare domain for recognition or surveillance. The two modules are not at all dependent on each other, but innovation depends on the high availability and low response time. For pattern recognition and expert system, HAR is a specific application base. The recognition part is done in two phases, Training and Recognition. Both of the following phases have the same steps; the training part contains information prior of any of the activities that are done, and the other recognition phase uses the training phase information to give highly accurate results. This results in the dependency of the recognition phase being very high in contrast to that of the training phase [10].

**Figure 1:**
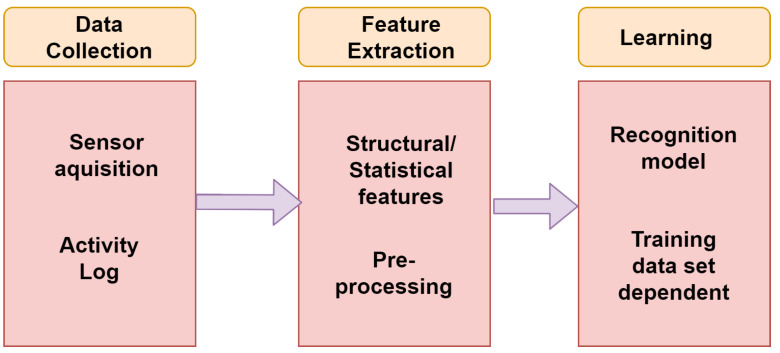
Learning phase of HAR.

**Figure 2:**
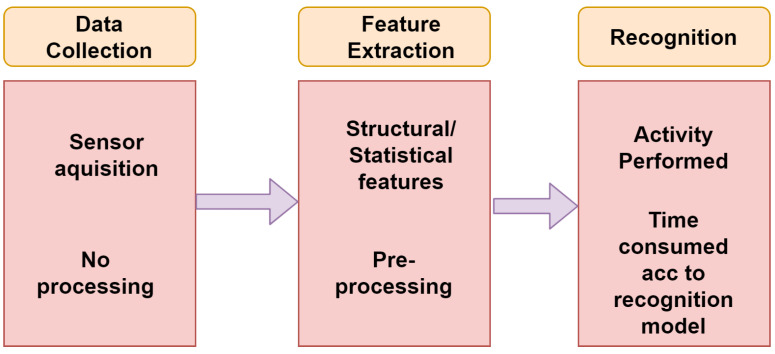
Recognition phase of HAR.

1. **Training or Learning Phase:** Recognition algorithm’s first step is training or learning phase. This phase is responsible for building the relationship between data and the activities, which is more clearly seen in Figure 1. Further are the following steps:
  - **Data Collection:** This step acquires systems data from all the sensors available. Sensor selection is based on the type of device for which the recognition system is made for. The following steps contains a log of activities of every activity performed with the type of activity, time, and the respective duration. This step works independently without any signal interference as all these will take place in the next following steps.
  - **Feature Extraction:** This step is fully concerned with the above step as it is dependent on the types of variables and kind of sensors involved in the data collection. It contains both statistical and structural features. Use of signal for any transformation comes under statistical feature extraction. With the help of structural feature extraction, interrelation and correlation among the signals can be found. The features could be mean, standard deviation, or correlation of a signal, and the transformations could be Fourier or Wavelet. In the following step, elimination of noise in the signal and range reduction of signal is done so that better features of any activity can be extracted.
  - **Learning:** The final step is the building of a recognition system learned from a set of data, activity log, and also from the extracted features so that the activities could be properly recognized. The following step is highly dependent on the training dataset. There are many recognition models built based on neural networks, fuzzy logic, and rule tree based on signal parameters, each having its own pros and cons. The best one can be selected based on complexity, resources available, and response time.
2. **Recognition Phase:** The last phase of the human recognition system is done with the help of the training phase that results from which the recognition of activity that can be performed better, as shown in Figure 2, described with the following steps:
  - **Data Collection:** This particular step obtains the data from all those sensors which are available for the process of recognition. Based on a device for which the recognition is made for, the process of sensor selection can be done. In the learning phase, prior knowledge of the activities is a must, but, in the recognition phase, there is not any prior knowledge of the activities performed, which is why no activity log is required. As discussed above, data collection can be done without any signal processing, and that rest all can be done in the next feature extraction step.
  - **Feature Extraction:** This step is fully concerned with the above step as it is dependent on the types of variables and kind of sensors involved in the data collection. It contains both statistical and structural features. Use of signal for any transformation comes under statistical feature extraction. With the help of structural feature extraction, interrelation and correlation among the signals can be found. The features could be mean, standard deviation, or correlation of a signal, and the transformations could be Fourier or Wavelet. In the following step, elimination of noise in the signal and range reduction of signal is done so that better features of any activity can be extracted.
  - **Recognition:** The final step is the speculation of the activity being performed with the help of the integrated data, and features can be extracted based on the prior steps by using recognition model. Depending on a recognition model, the possible activities can be performed with accuracy percentage. This is the most time-consuming step of the human recognition system according to which model is being selected and resources available.

## 4. Deep Learning in HAR

**Figure 3:**
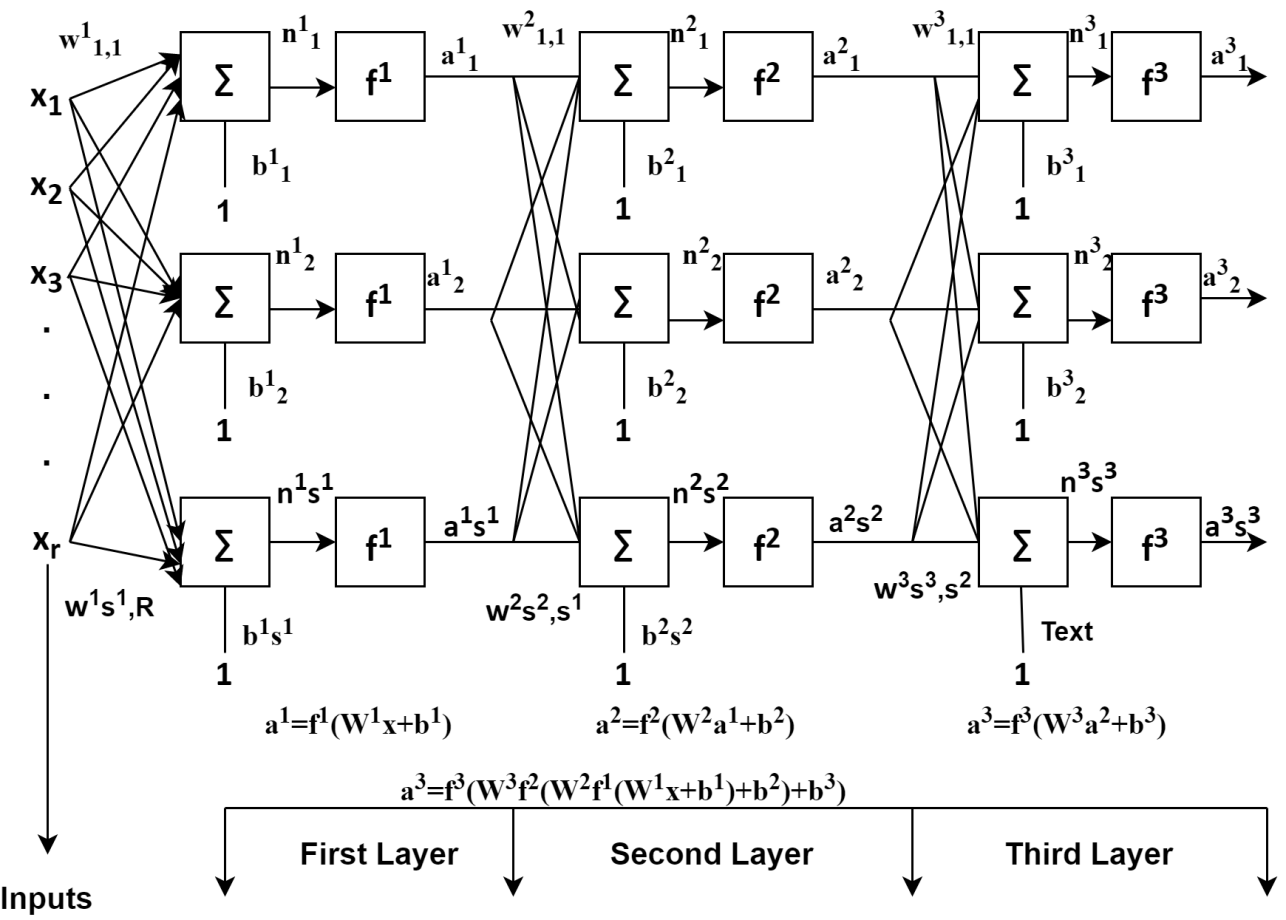
Feed forward neural network.

Deep learning is a field that is currently emerging. This is because automatic feature extraction can be performed. In the previous works, with the help of sensing devices, data was collected, and results analysis and development of effective HARs takes place [13]. One-dimensional Convolutional Neural Network (CNN) was proposed for the identification of walking, running, and no motions by using a 3-axis accelerometers in smartphones carried by the users. Input for this was the acceleration data of x,y,z for neural networks, and accuracy of about 92.7% achieved. In Reference [6], for the feature learning process, it was conducted automatically through CNN, thus having capabilities of both accuracy and complexity. Reference [17] came up with the deep CNN models for the five daily activities, such as walking, walking upstairs, walking downstairs, sitting, and sleeping, and took the data from both accelerometer and gyroscope of the wearable device and achieved accuracy of about 96.4%. The classifiers discussed above suffer from a problem, such as increase in computation power, making it hard to deploy. The activities discussed are concealed as a series of readings the sensors can take in a particular time T [21]. Some routine activities are being identified in Reference [22], such as sitting, walking, standing, and squats, using deep learning achieved an accuracy of about 82%.Techniques of machine learning were not able to capture temporal co-relations between inputs. The classifier CNN can address the issue [17]. RNN, which is a deep learning model, also known as feed forward network and shown in Figure 3, is also used for the sequential activities LSTM, being one of the further techniques of RNN that can address the issue and gained the popularity. These networks are very much capable of capturing sequential data, along with temporal information.

## 5. Public Datasets

Various HAR public datasets are sensor-based [23] and vision-based [24].The recently generated datasets for Human Activity Recognition are as follows:

**Sensor-based Dataset:** Some inertial sensors for smartphones for tracking the various physical activities, such as accelerometers and gyroscope. Participation of thirty subjects took place with a belt through which smartphones have been attached. Six basic and traditional activities are part of this dataset. There are more open datasets available in Reference [25]. The particular dataset has been generated using smartwatches, smartphones, and smart glasses, such as accelerometer, heart rate, gyroscope, step counter, etc.

**Vision-based Dataset:** The dataset, namely COIN, is created with the routine activities of daily life videos associated to vehicles, tools, etc. It is general video analysis, and all containing videos were collected from YouTube [26]. Another dataset is known as VIRAT, having tedious occurrences, such as communication among group of different people, means of transport and facilities with the number twelve activities can be taken as single person events, the interaction between people and means of transport, and, lastly, person and facility events [27], and is made up of surveillance video. In addition, for the physical activities of UCI-HAR dataset [28], along with the sample and the subject, refer to Table 1.

The dataset used here for the method proposed in this is the [14] collection of data from various volunteers of 19–48 age group having a smartphone. The phone having both accelerometer and gyroscope can be used to collect the signals’ acceleration and angular velocity at a sampling rate of 50 Hz. Median filter is used to process the signals and a low pass filter with 20 Hz cut-off frequency. From input samples, calculation of various parameters, such as mean, standard deviation, median, largest values, smallest value, signal magnitude area, average sum of the squares, signal entropy, auto-regression coefficients, correlation coefficient, largest frequency component, frequency signal weighted average, frequency signal skewness, frequency signal kurtosis, energy of a frequency interval, angle between two vectors, etc., takes place. After feature extraction, 561 features are obtained in total. The 30–70% rule is followed at the time of division of the two groups as 70% of the data of the dataset is used in training and remaining 30% for testing. There is a total absence of raw data. Therefore, instead of raw data, the dataset’s pre-processed version was made available for the use. The pre-processing process has some steps to follow, such as:

1. Use of noise filters for both accelerometer and gyroscopes pre-processing.
2. With 50% of overlapping, data can be segmented into fixed windows of 2.56 s, as well as 128 data points.
3. Accelerometer data is partially segmented into gravitational which is also called total and body motion components.

On Window data application of feature engineering is the main step, through which copy of that data with these engineered features made available to use. From each window, extraction of a number of features related to frequency and time can be obtained. Five hundred and sixty-one element vectors are the result of that features. The dataset can be split into 70% for the training purpose and 30% sets for testing purpose based on data for the subjects being used, for example, 21 subjects for training and 9 for testing as shown in Table 2.

Total number of samples taken: 1,576,645 Total number of subjects taken: 30.

## 6. Lightweight Deep Learning Classifier Hybrid Model

To capture spatial-temporal information in sequential pattern, both CNN and LSTM are being used for recognition with score fusion. Along with this, the experimental results shows the better score fusion between Convolutional network and Long-Short term based memory (LSTM) as compared to LSTM-LSTM.Using this model can not only improve the predictive accuracy but also help in reduction of the models complexity, along with need for advanced feature engineering. This CNN-LSTM can be considered as spatially and temporally deep. This hybrid model effectively covers the problems of image description, video description, and activity recognition. We have proposed the hybrid model for human activity recognition. The process of how the model is proposed is based on the following.

### 6.1. Deep Learning Classifier-Convolutional Neural Network (CNN)

**Figure 4:**
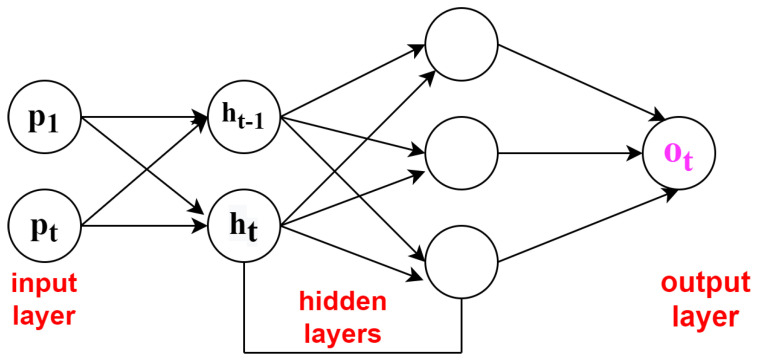
Lightweight deep learning classifier-convolutional neural network.

CNN is considered to be the main algorithm for conducting feature extraction and recognition of six daily activities. In this section, for the recognition of human daily activities dataset, building a one-dimensional convolutional neural network model takes place. These can be developed by taking both acceleration and gyroscopic data based on single dimensional serial data for any human activity recognition. The series of observations obtained are used for mapping of the internal features into different types of activities and also used to extract the features. The main advantage of using this deep learning classifier Convolutional network is that these networks are able to directly learn from the series of raw data, and expertise for the manual input features is not at all required. This model also has the capability to learn internal representation of serial-time data and achieves better performance to fit on a best feature engineered model. A lightweight deep learning classifier CNN is shown in Figure 4, in which $p_{1}$ and $p_{t}$ are the input layers, $h_{t}-1$ and $h_{t}$ are the hidden layers, and $o_{t}$ is the output layer.The basic architectures of feed forward network and recurrent neural networks are shown in Figure 3 and Figure 4. The feed forward network gives accuracy of about 97.4%, and RNN individually is 95.5% accurate [16].

Total acceleration, body acceleration, and body gyroscope are the three types of signals available in the raw data. Each came up with the three axes of data results in the total of nine variables taking place at each step. The serial data is split into a window overlapping of 2.65 s of data, or 128 time steps. The data window corresponds with windows of engineered features also known as rows in the prior section. The elements in each row of data have 128 × 9 or a total of 1152 elements. This can be seen as a bit less than doubling the size of the aforementioned 561 element vectors, and there could also be chances of the same data.

### 6.2. Preparation of Data

The main datasets body acceleration, body gyroscopic, and total acceleration have the values of −1, 1. It is still unclear whether the value of data is for per-subject or for all of the subjects. Observations for a model can be standardized prior for more improvement and, of course, for the better results. The method known as Gaussian method can be used for the shifting of the distribution for each variable and results in the parameters, such as mean of value 0 and standard deviation of value 1. The way to check each variables distribution in training dataset is to plot a histogram of each variable. By doing this, a problem of splitting of data into 128 time step window data with overlapping of a number 50 exists. As a result, for the fair results of distribution of data, the points should be remembered as including the removal of same or overlapped observations and also data windowing should be eliminated.

### 6.3. Recurrent Neural Network: LSTM

**Figure 5:**
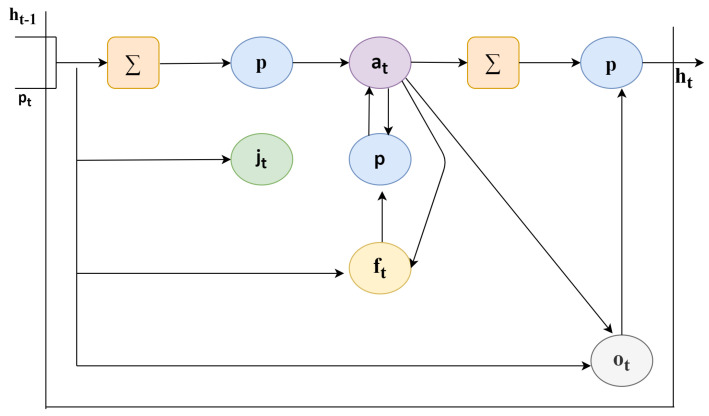
Lightweight deep learning classifier-long short term memory cell.

As discussed, the convolutional model is able to work on single image and change the image from input pixels into vector or matrix representation. For the feature extraction process, an existing pre-trained model use off CNN is fixed. The major point of discussion here is that CNN may not be trained but training can be provided with the help of back propagating error from the Long-Short Term Memory deep learning classifier across CNNs multiple input images. A typical LSTM cell is shown in Figure 5. The LSTM cell consists of variety of parameters and gates to control behavior of each memory cell. Each cell state is controlled by the activation functions of gates. To different types of gates available the input values are fed to: forget gate (f), input gate (i), output gate (o), and activation vector (c).

(1) $$j_{t}=\in_{j}\left(w_{pj}p_{t}+w_{hj}h_{t-1}+w_{aj}a_{t-1}+bj\right),$$

(2) $$f_{t}=\in_{f}\left(w_{pf}p_{t}+w_{hf}h_{t-1}+w_{af}a_{t-1}+bf\right),$$

(3) $$a_{t}=f_{t}a_{t-1}+j_{t}\in_{a}\left(w_{pc}p_{t}+w_{ha}h_{t-1}+ba\right),$$

(4) $$o_{t}=\in_{o}\left(w_{po}p_{t}+w_{ho}h_{t-1}+w_{ao}a_{t-1}+bo\right),$$

(5) $$h_{t}=o_{t}\in_{h}\left(a_{t}\right),$$

where ($w_{pj}$, $w_{hj}$, $w_{aj}$, $w_{hf}$, $w_{af}$, $w_{pa}$, $w_{ha}$, $w_{po}$, and $w_{ao}$) are weights ($w_{pj}$ = input-input weight, $w_{hj}$ = hidden input weight, and so on), and $b_{j}$, $b_{f}$, $b_{a}$, and $b_{o}$ are the bias weights.

Both the cases for each time step consist of one CNN model and series of LSTM models. To each input image, CNN can be applied and can be passed on output of each to the LSTMs input image just as a single step. The results can be in the hands by folding up of CNN’s input architecture of one or can be more layers in a Time Distribution model. The same results can be obtained by applying either the same layer or layers a multiple number of times. To work on the multiple input time steps gives a series of image interpretations features to the LSTM model.

### 6.4. Working of CNN-LSTM

**Figure 6:**
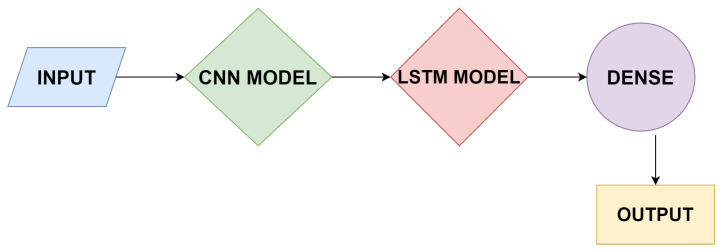
Working of CNN-LSTM.

For feature extraction on the input layer, the use of convolutional neural network is good option to work upon with the combination of LSTMs so that the prediction of sequence can be done. The working of CNN-LSTM is shown in Figure 6. The input in the form of human activities would be taken, and the next step is to apply the CNN model, then the LSTM model, and then at last the dense layer to check whether both the models are working properly or not. After that, the desired output will be generated. The combination of both the classifiers was built for the prediction of time sequence problems, and textual descriptions can be generated from the concatenation of images, for example, videos. Specifically, the problems of **Activity Recognition** produce a textual explanation of an activity displaying in a chain of images. **Image Description** produces a textual explanation of a single image. **Video Description** produces a textual explanation of a chain of images.

**Figure 7:**
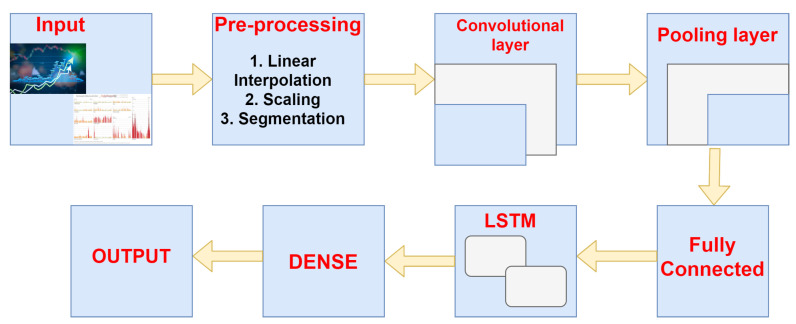
Efficient and lightweight deep learning model.

The architecture followed here is known as a Long-term Recurrent Convolutional Network or LRCN model, additionally referred to as “CNN LSTM”, which means LSTMs that use a CNN as a front end in the following process. The combination of CNN-LSTM gives an efficient and lightweight deep learning model, as shown in Figure 7. The input part will track the human activities in sequence, after which the pre-processing of the sequence data using linear interpolation, scaling, and segmentation, and then using CNN approach, and a convolutional layer will pass the data to pooling layer and then to the fully connected layer then the LSTM will pass the processed data by deciding whether to keep or forget the information. Then, the dense layer will tell whether the proposed architecture is working in an appropriate manner, for example, to check whether the CNN model is extracting the valuable features and LSTM model for the interpretation of features in sequential manner. Then, finally, the recognition of the human activities takes place. This model is used for producing textual explanation of images, as well. The main key point to use the CNN that on any image classification task the pre-training is quite challenging but can be reallocated as a feature extractor also for the production of various caption problems. The various applications of the following model is to solve the natural language processing(NLP) and also speech recognition related problems where the CNN classifier is used for extracting the features and LSTMs are being used for thr input data. The CNN-LSTM has various problems which are being discussed below:

- The input part having the spatial structure, such as one-dimensional and two-dimensional images, having structure or pixels in the form of combination of words, paragraph, and can be a document file.
- The input part also consists of temporal structure which can be in form of words in an image or video therefore generating an output in the same form is a challenging task.

## 7. Results Analysis and Discussions

In this section, we have described various parameters along-with results achieved after applying the lightweight model for Human Activity Recognition.

### 7.1. Performance Metrics

The performance metrics described below depends on how efficient is the dataset being used. And the efficiency of the dataset depends on the following metrics, which can be described as follows, and also Table 3, which is a confusion matrix that elaborates the values of physical activities with respect to the dataset used having accuracy, precision (Prec), recall (Rec), and f1-score values.

- **Accuracy:***“It is defined as the number of correct predictions over total number of True Positive predictions by the model”,*
  (6) $$Accuracy=\frac{TP+T}{TP+TN+FP+F}.$$
  *where TP = True positive class prediction, TN = True Negative class prediction, FP = False positive class prediction, FN = False negative class prediction*.
- **Recall:** *“It is defined as Number of true predictions over actual number of true predictions made by the model”.*
  (7) $$Recall=\frac{1}{C}\left(\sum_{c=1}^{C}\frac{TP_{c}}{TP_{c}+FN_{c}}\right).$$
- **Precision:** *“It is the number of actual true predictions over total true predictions made by the model”.*
  (8) $$Precision=\frac{1}{C}\left(\sum_{c=1}^{C}\frac{TP_{c}}{TP_{c}+FP_{c}}\right).$$
- **F1 Score:** *“It is two times the precision and the recall to the sum of precision and recall”.*
  (9) $$F1Score=\frac{2XPrecisionXRecall}{Precision+Recall}.$$

### 7.2. Gaussian Graph of HAR

**Figure 8:**
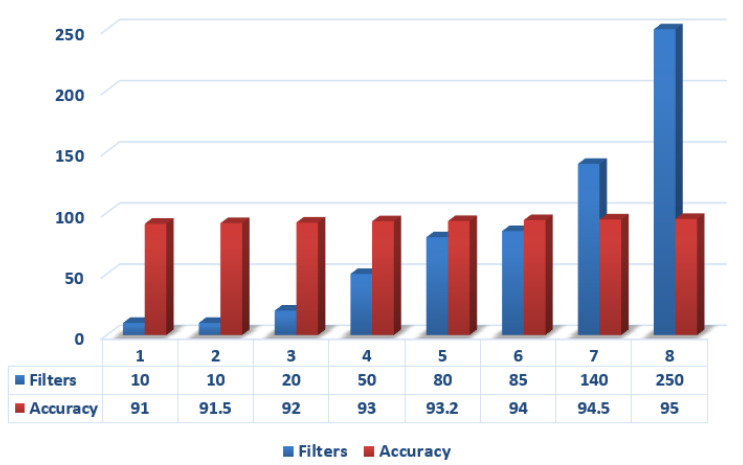
Comparison of deep learning classifiers in different size of filters.

**Figure 9:**
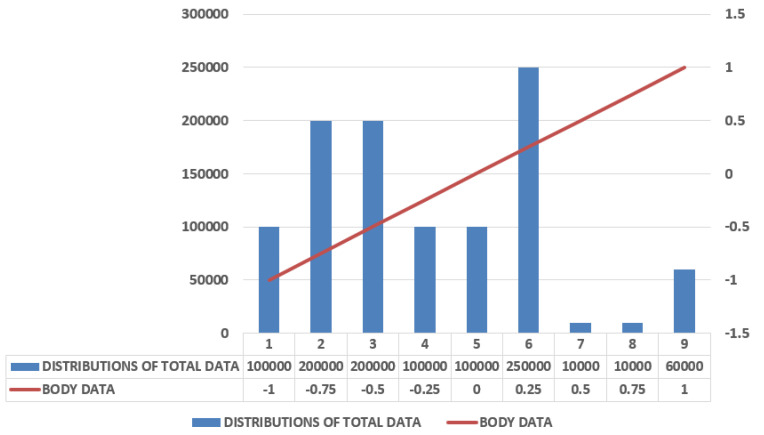
Depiction of Gaussian data with respect to data of HAR.

The experiment done above can also be done with the help of machine learning classifier SVMs, that is, support vector machines using a smartphone only, and gave the result of accuracy of about 89% based on the testing dataset. Figure 8 shows that the reading between body data with in comparison to the distributions of total acceleration data, and Figure 9 depicts the Gaussian Data with respect to HAR, showing the results between the distribution of the total data taken to that of the body data.

### 7.3. Experimental Requirements

The following model can be achieved by performing on Raspberry Pi3 with 4x ARM Cortex A-53, 1.2 GHz processor, and 4 GB RAM. The model for human activity recognition is implemented in Python 3.8.8 and Tensorflow 1.7. Some of the basic steps took part in experimentation are discussed one-by-one below. Figure 8 shows the results of accuracy with that of the filters. A function known as evaluate model, which picks both training and testing datasets and is able to fit a model based on training, then evaluates on the test data and, finally, gives output with the models performance estimation.

### 7.4. Steps to Load the Data

The Keras deep learning library is used by deep learning convolutional neural network for the implementation is the initial step to start upon. The three-dimensional input, such as samples, time steps, and features, are also required for further steps. The data is hereby used for loading of the one sample in one window having time series data with 128 time steps, along with 9 features. The output obtained is a 6-element vector having a probabilistic approach for a window that belongs to each of 6 activities being discussed in the following experiment. All the steps individually matter with both input and output dimensions, which are mandatory for model fitting, and then, using training dataset, the features can be extracted.

### 7.5. Configurations Affecting the Model

**Figure 10:**
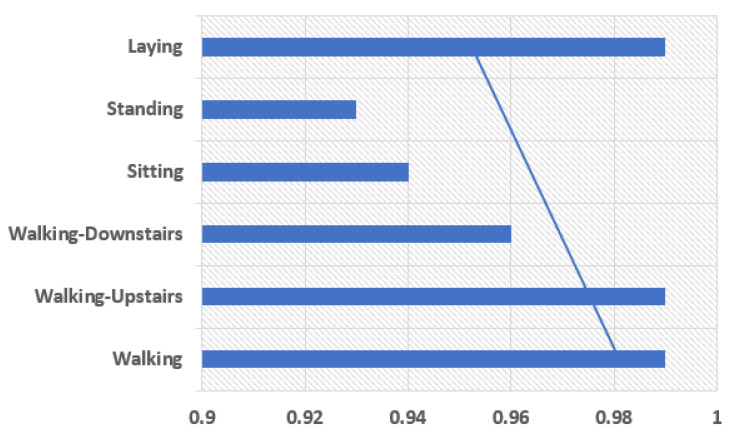
Precision.

**Figure 11:**
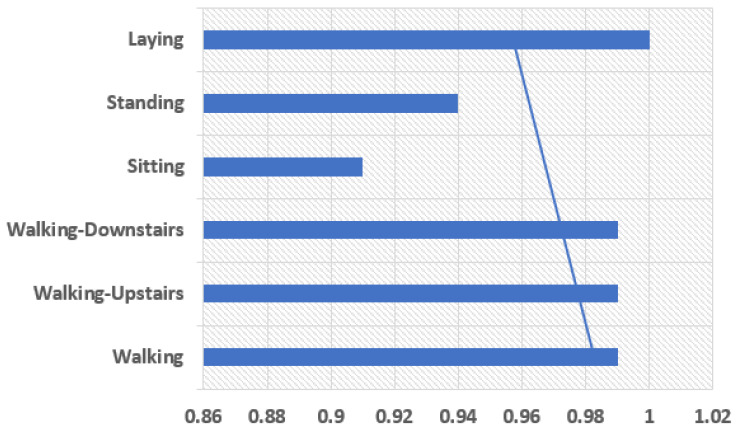
F1 Score.

The model is comprised of the two one-dimensional CNN layers with a dropout layer for the regularization process followed by a pooling layer. The CNN layers in a group of two can be defined so that the model can learn the features fast from the input data. Though it is known that CNN learns very fast the work of dropout layer is to make the learning process slow so that the model can be able to work accurately. The work of pooling layer is the reduction of learning features to 1/4 their size by considering only the most important elements. Then comes the learned features that are being flattened to one of those long vectors and before output layer predicts any result the features can firstly be passed through a fully connected layer. The use of fully connected layer is that it will be able to provide a buffer for the prediction process, a storage between learned features and output. A standard configuration of 64 parallel feature maps and a kernel size of 3 is the major requirement of this model. The number of times the processing of input takes place is the feature mapping, and the number of input steps should be considered as sequence required for input in kernel size used to read or processed the learning features. For the network optimization, an efficient method known as gradient descent is used. The requirements for a model to be fit for a fixed number of epochs 10, batch size of 32 samples, exposure of 32 windows of data but before the models weight updation. The graph between human physical activities with precision values and the f1 score the results can be shown in Figure 10 and Figure 11.

The precision parameter shows the more precise results in Walking, Walking Upstairs, and Laying. The F1-Score values show the best results in Walking, Walking Upstairs, Walking Downstairs, and Laying. Discussing the following parameters makes the model fit to use. Once the model is fit, it can be evaluated on the test dataset and then return with the accuracy of the fit model on the test dataset. Our model is the best fit giving the best accuracy of 97.89%. Table 4 shows the comparison of our proposed model’s accuracy with that of the existing related work done.

**Figure 12:**
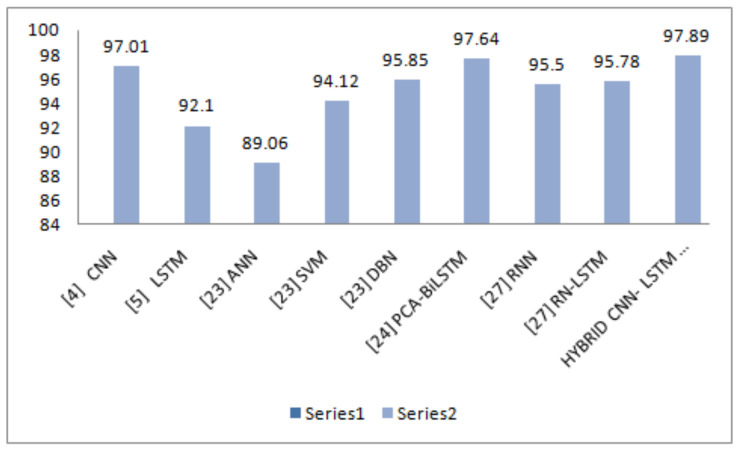
Comparison of accuracy of proposed model with state-of-the-art models.

In addition, the various deep learning classifiers with their following accuracies are covered in the graph, as shown in Figure 12, which shows that our proposed model is 97.89% accurate.

## 8. Conclusions

The lightweight model of a deep neural network with CNN-LSTM for HAR was proposed in this paper. The main focus of the weighing parameters is on fully connected layers. To capture the different portions of the image while walking, laying, upstairs/downstairs’ movements, etc., filtering technique and, for network optimization, an efficient method known as gradient descent are used. The requirements for a model are to be fit for a fixed number of epochs 10, batch size of 32 samples, and exposure of 32 windows of data, but before the models weight updations’ implementation. The Keras deep learning library for deep learning convolutional neural networks is used for the implementation purpose. The model required a three-dimensional input, such as samples, time steps, and features, being loaded, where each sample has one window of the time sequential data with 128 time steps containing 9 features. The proposed model has shown the better accuracy over ANN, LSTM, PCA-BiLSTM, FFNN, DBN, SVM, RNN-LSTM, CNN, and RNN using Gaussian Method. Achieving more accurate results for different types or combinations of deep learning techniques on various types of datasets is the further area of investigation.

## Figures and Tables

**Table 1:** Comparative study of existing work.

Author Name (Year) [Reference No.]	Objective	Proposed Methodology	Pros	Cons
Wang et al. (2016) [11]	A feature selection approach for activity recognition and to reduce the smartphone power consumption	A Smartphone-based activity recognition framework is being proposed	Use of built-in accelerometer and gyroscope in smartphones	The power of the triaxial accelerometer and gyroscope in activity recognition still needs more research.
Agarwal et al. (2020) [16]	Lightweight Deep learning model for human activity recognition	Uses RNN-LSTM approach	Less computational power and easy deployment on edge-devices	Can be extended to support multi-sensor data.
Zebin et al. (2016) [17]	Human Activity Recognition with Inertial sensors using deep learning approach	Use of Convolutional Neural Network	Achieved best results as compared to other methods	For multiple sensors use of CNN-LSTM sequence classifier must be studied.
Chen et al. (2016) [9]	Network for mobile human activity recognition	Long-short term memory approach for human activity recognition	Use of tri-axial accelerometers data	Can be extended for multi-sensor data.
Lee et al. (2017) [13]	Using tri-axial accelerometers in smartphone recognition of physical activities	Convolutional Neural Network method for HAR being proposed	Achieved accuracy of about 92.71%	Improvement in recognition of human activities needs more accurate results.
Hasan et al. (2018) [18]	A Robust Human Activity Recognition System Using Smartphone Sensors and Deep Learning	Smartphone inertial sensors-based approach	Compares different deep learning models	More efficient and complex activity recognition in real-time environments.
Aljarrah et al. (2019) [14]	Human Activity Recognition tasks based on wearable sensors data	PCA-BiLSTM approach	Achieves accuracy of about 97.64	Not suitable for real-time environments.
Xia et al. (2020) [15]	Convolutional Neural Network with Long Term Short Memory using three types of datasets and compares accuracy with each of the dataset	CNN-LSTM architecture is being proposed	Achieved different accuracies based on the different datasets	Performance based on different parameters need to be evaluated further.

**Table 2:** Activities of UCI-HAR.

Activity	Samples	Percentage (%)
Walking	614,892	39%
Walking Upstairs	472,994	30%
Walking Downstairs	189,198	12.2%
Sitting	126,132	8.9%
Standing	96,176	6.1%
Laying	78,833	5%

**Table 3:** Confusion matrix of UCI-HAR.

Activities	Walking	Walking Upstairs	Walking Downstairs	Sitting	Standing	Laying	Prec	F1	Rec
Walking	489	0	7	0	0	0	0.99	0.99	0.99
Walking Upstairs	4	444	23	0	0	0	0.99	0.99	0.96
Walking Downstairs	5	1	414	0	0	0	0.96	0.99	1.00
Sitting	0	11	0	432	0	0	0.94	0.91	0.93
Standing	1	0	0	0	419	0	0.93	0.94	0.93
Laying	0	27	1	0	112	510	0.99	1.00	0.99

**Table 4:** Comparison of accuracy of proposed model with state-of-the-art models.

Reference	Model	Accuracy (%)
[12]	Convolutional Neural Network (CNN)	97.01%
[9]	Long-Short Term Memory (LSTM)	92.1%
[18]	Artificial Neural Network (ANN)	89.06%
	Support Vector Machine (SVM)	94.12%
	Deep Belief Network (DBN)	95.85%
[14]	Principle Component Analysis- Bidirectional Long-Short Term Memory (PCA-BiLSTM)	97.64%
[16]	Feed Forward Convolutional Network (FFCN)	97.64%
	Recurrent Neural Network (RNN)	95.5%
	Recurrent Neural Network-Long-Short Term Memory (RNN-LSTM)	95.78%
Proposed Model	Hybrid Convolutional Neural Network-Long-Short Term Memory (Hybrid CNN-LSTM)	97.89%

## Data Availability

Not applicable.
